# TDO2 overexpression correlates with poor prognosis, cancer stemness, and resistance to cetuximab in bladder cancer

**DOI:** 10.1002/cnr2.1417

**Published:** 2021-06-07

**Authors:** Quoc Thang Pham, Daiki Taniyama, Shintaro Akabane, Kenji Harada, Takashi Babasaki, Yohei Sekino, Tetsuraro Hayashi, Naoya Sakamoto, Kazuhiro Sentani, Naohide Oue, Wataru Yasui

**Affiliations:** ^1^ Department of Molecular Pathology Hiroshima University Graduate School of Biomedical and Health Sciences Hiroshima Japan; ^2^ Department of Pathology University of Medicine and Pharmacy at Ho Chi Minh City Ho Chi Minh City Vietnam; ^3^ Department of Urology Hiroshima University Graduate School of Biomedical and Health Sciences Hiroshima Japan

**Keywords:** bladder cancer, cancer stem cell, cetuximab, EGFR, spheroid formation, TDO2

## Abstract

**Background:**

Bladder cancer (BC) is the 10th most common cancer in the world. BC with muscle invasion results in a poor prognosis and is usually fatal. Cancer cell metabolism has an essential role in the development and progression of tumors. Expression of tryptophan 2,3‐dioxygenase (TDO2) is associated with tumor progression and worse survival in some other cancers. However, no studies have been performed to uncover the biofunctional roles of TDO2 in BC.

**Aim:**

This study aim to investigate the clinicopathologic significance of TDO2 in BC.

**Methods and results:**

TDO2 expression was evaluated by qRT‐PCR and immunohistochemistry in an integrated analysis with the Cancer Genome Atlas (TCGA) and other published datasets. TDO2 overexpression was significantly associated with T classification, N classification, and M classification, tumor stage, recurrence, and basal type, and with the expression of CD44 and aldehyde dehydrogenase 1 (ALDH1) in BC. High TDO2 expression correlated with poor outcome of BC patients. Using BC cell lines with knockdown and forced expression of TDO2, we found that TDO2 was involved in the growth, migration, and invasiveness of BC cells. Moreover, TDO2 was found to be crucial for spheroid formation in BC cells. Importantly, TDO2 promoted BC cells resistance to cetuximab through integration of the EGFR pathway.

**Conclusion:**

Our results indicate that TDO2 might take an essential part in BC progression and could be a potential marker for targeted therapy in BC.

## 1 INTRODUCTION

Bladder cancer (BC) is the 10th most leading cancer in the world.^1^ In Japan, BC is the 8th most leading cancer in men and the 11th leading cancer in women.^2^ Almost 70%–80% of the newly diagnosed BC patients present with non‐muscle‐invasive or early‐invasive disease and the rest of patients with muscle‐invasive bladder cancer (MIBC).^3^ Radical cystectomy is the current standard treatment for MIBC patients without metastasis.^4^, ^5^ Although recent innovative gains in the knowledge of new molecular classifications of BC and some remarkable therapeutic strategies have been investigated, the outcome of patients with MIBC remains poor.^5^ Therefore, it is necessary to find new biomarkers for therapeutic targets in BC.

Currently, tumor cell metabolism has shown a pivotal role in tumor development and tumor progression and has become a therapeutic target for cancer treatment.^6^, ^7^ It was reported that tryptophan metabolites are highly detected in the urine of BC patients.^8^ The kynurenine (Kyn) pathway, which included two main enzymes: indoleamine 2,3‐dioxygenase 1 (IDO1) and tryptophan 2,3‐dioxygenase (TDO2), takes up almost 95% of tryptophan metabolism.^9^ IDO1 is expressed in a variety of tissues, while TDO2 is primarily expressed in the liver.^10^ TDO2 overexpression has been described in many cancers including glioblastoma, breast cancer, and lung cancer.^10^, ^11^, ^12^ Overexpression of TDO2 enhanced tumor cell survival and associated with worse outcomes in patients with these tumors.^10^, ^11^, ^12^ We previously reported that the expression of TDO2 was linked with tumor progression and poor prognosis and cancer stem cells (CSCs) in esophagus squamous cell carcinoma.^13^ CSCs, accounting for around 1% of solid tumor cells, are known to be more resistant to conventional treatments than other cancer cells within a tumor.^14^, ^15^ However, the clinicopathologic significance of TDO2 in BC remains unknown.

Here, we investigated the expression of TDO2 in BC tissues and studied the relation of TDO2 with the clinicopathologic features and prognosis of BC. We also explored the biofunctional roles of TDO2 in progression, cancer stemness, and drug resistance using BC cell lines with RNA interference (RNAi) knockdown and forced expression of TDO2.

## 2 MATERIALS AND METHODS

### 2.1 Tissue samples

A total of 59 primary tumors were obtained from 50 patients diagnosed with MIBC who underwent radical cystectomy at National Hospital Organization Kure Medical Center and Chugoku Cancer Center (Kure, Japan) and from nine patients at Hiroshima University Hospital (Hiroshima, Japan). Informed consent was obtained from each patient. This study was approved by the Ethical Committee for Human Genome Research of Hiroshima University and the Ethics Committee of Kure Medical Center and Chugoku Cancer Center. The TNM classification system was used to determine tumor staging.^16^

We used 9 BC samples and 10 types of normal tissue samples for qRT‐PCR. Tumor tissues and nonneoplastic tissues were surgically removed, immediately frozen in liquid nitrogen, and stored at −80°C. Normal tissue samples were purchased from Clontech Laboratories, Inc. (Mountainview, CA), including the brain (catalog no. 636530), heart (636532), lung (636524), stomach (636578), small intestine (636539), colon (636553), liver (636531), kidney (636529), skeletal muscle (636547), and bladder (636542).

For immunohistochemical (IHC) investigation, we use formalin‐fixed, paraffin‐embedded tissues. Two tumor blocks in each patient, including the tumor and the tumor with nonneoplastic epithelial tissue, were evaluated by IHC staining.

### 2.2 Analysis of TCGA and public datasets

An online analytical web‐based tool, https://xenabrowser.net/, was conducted to evaluate the mRNA expression of TDO2. Bladder urothelial carcinoma (BLCA) was selected, and TDO2 was selected as the target gene for further analysis. To investigate the relationship between TDO2 and other genes associated with the basal or luminal subtype of BC, we downloaded the public dataset (GSE31684 and GSE48277) from Gene Expression Omnibus (GEO) database, https://www.ncbi.nlm.nih.gov/geo/.

### 2.3 qRT‐PCR analysis

Total RNA was isolated from cells pellet and frozen tissue by using ISOGEN (Nippon Gene, Toyama, Japan). A total of 1 μg RNA was used to synthesis cDNA by the PrimeScript first strand cDNA Synthesis Kit (Takara Bio, Shiga, Japan). PCR was performed with a CFX Connect real‐time PCR detection system (Bio‐Rad) using the SYBR Green PCR Core Reagents Kit (Applied Biosystems; Thermo Fisher Scientific, USA). The 2^−ΔCT^ method was used to calculate the relative expression levels, in which ΔCT is the difference in threshold cycle (CT) values between the target gene and Actin Beta (ACTB) served as an internal control. The primer sequences are listed in Data S1.

### 2.4 Immunohistochemistry and evaluation

The IHC staining procedure was carried out as previously described on 3 μm thick sections.^13^ Primary antibody mouse polyclonal anti‐TDO2 antibody (1:250; catalog no. H00006999‐B01P, Abnova, Taipei, Taiwan), mouse monoclonal anti‐GATA3 (1:100, catalog no. ACR405A, Biocare Medical, Pacheco, CA), and mouse monoclonal anti‐34βE12 (1:200, catalog no. M063029‐2, Dako, USA) were used to detect protein expression. The expression of TDO2 was evaluated by the expression score, which included the intensity (1+, 2+, 3+) and the percentage (from 0% to 100%) of detected tumor cells. The expression of GATA3 and 34βE12 was evaluated by the percentage of staining cells. IHC staining of CD44 and ALDH1 were carried out as previously described.^17^

### 2.5 Western blot analysis

Cell pellets were lysed in Ripa buffer (50 mM Tris, pH 7.4, 125 mM NaCl, 0.1% NP40, 5 mM EDTA, and protease inhibitor cocktail [cOmplete, Roche]).^18^ Western blot procedures were carried out as previously described.^18^ The following primary antibodies were used: anti‐TDO2 antibody (H00006999‐B01P, Abnova, Taipei, Taiwan), Anti‐Erk (9102), anti‐Phospho‐p44/42 MAPK (pErk1/2, 9101), anti‐Akt (9272), anti‐phospho‐Akt (pAkt, 4060), Vimentin (D21H3), and E‐Cadherin (24E10) antibodies (Cell Signaling Technology, Danvers, MA). Immunocomplexes were detected with an ECL Western Blot Detection System (Amersham Biosciences, Little Chalfont, Buckinghamshire, UK). β‐Actin (Sigma‐Aldrich, St. Louis, MO) was served as an internal control.

### 2.6 Cell lines

Seven BC cell lines (T24, RT112, 253 J‐BV, KMBC2, UMUC3, UMUC6, UMUC13) were grown in RPMI‐1640 (Nissui Pharmaceutical Co., Ltd., Tokyo, Japan) containing 10% fetal bovine serum (BioWhittaker, Walkersville, MD). Cells were cultured in a humidified incubator at 37°C with 5% CO_2_. T24 and KMBC2 were purchased from the Japanese Collection of Research Bioresources Cell Bank (Osaka, Japan), and the other cell lines were kindly provided by Prof. Peter C. Black (Department of Urologic Sciences, Vancouver Prostate Centre, University of British Columbia, Vancouver, Canada). Authentication of the cell lines was confirmed by short tandem repeat (STR) profiling.

The primer sequences for qRT‐PCR, RNA interference (RNAi), expression vector, proliferation assays, cell migration assays, invasion assays, spheroid colony formation assays, and statistical methods are presented in Data S1.

## 3 RESULTS

### 3.1 TDO2 expression in BC

**FIGURE 1:**
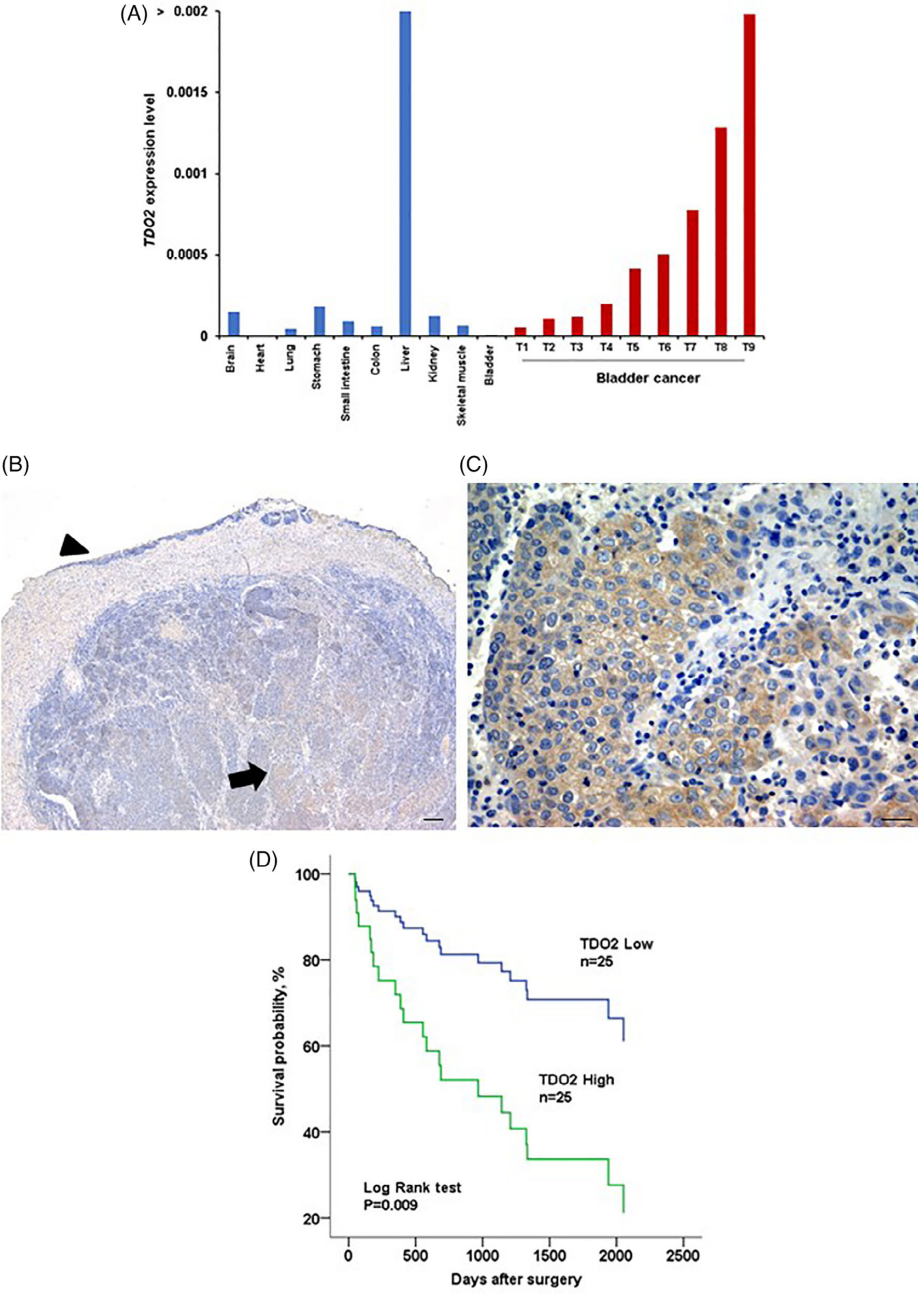
TDO2 expression in BC tumors. (A) TDO2 expression in 10 types of normal tissue samples and 9 BC tissue samples by qRT‐PCR. (B, C) IHC staining of TDO2 in BC tumors. (B) Normal urothelium showed weak cytoplasmic staining (arrowhead); tumor cells presented strong cytoplasmic staining with TDO2 (arrow). Scale bar, 100 μm. (C) Strong cytoplasmic TDO2 staining was observed in tumor cells. Scale bar, 10 μm. (D) Survival analysis of BC patients with high and low TDO2 expression by Kaplan–Meier method

We investigated the expression of TDO2 in BCs using the TCGA BLCA dataset. As shown in Figure S1a, TDO2 expression in BC tissue was significantly higher than that in normal bladder tissue. We evaluated TDO2 mRNA level in 10 kinds of normal tissue samples and 9 BC samples by qRT‐PCR analysis (Figure 1(A)). The TDO2 mRNA level was highest in the liver compared with that in other normal tissues. BC samples highly expressed TDO2 than normal tissues excepted liver tissue (Figure S1c).

Next, we conducted immunohistochemistry on the 50 BC tissue samples. Figure 1(B) shows that normal urothelium showed no staining or weakly staining for TDO2. Conversely, the expression of TDO2 was increased in BC and was detected in the cytoplasmic of tumor cells (Figure 1(C)). BC tissues showed heterogeneousness of TDO2 immunoreactivity. The TDO2 expression scores were calculated as detailed in the Methods section, and immunoreactivity images are shown in Figure S2a. The invasive areas and the central or superficial areas presented no difference in TDO2 expression score.

We further used ROC analysis to determine the cut‐off value for the TDO2 expression score that correlated with clinicopathologic features (Figure S2b–e). The cut‐off point of the expression score for T, N, and M classification, and stage was determined to be 75 by using the Youden index.^19^ TDO2 expression was defined as high if the expression score was above the cut‐off value and as low if the score was equal to or below the cut‐off value. Next, we investigated the correlation between TDO2 expression, divided by the cut‐off point, and the clinicopathologic characteristics of the 50 BCs (Table 1). High TDO2 expression was significantly related to advanced T (*p* = .002), N (*p* = .037), M classification (*p* = .037), tumor stage (*p* = .002), and recurrence status (*p* = .002). These data suggested that TDO2 might have a pivotal part in the progression of BC.

**TABLE 1:** Correlation between TDO2 expression and clinicopathologic features in bladder cancer

		TDO2 expression, *n* (%)		*p*‐value^a^
		High	Low	
Histological grade	Low grade	1 (20.0)	4	.157
	High grade	24 (53.3)	21	
T classification	T1/2	3 (18.8)	13	.002
	T3/4	22 (64.7)	12	
N classification	N0	13 (39.4)	20	.037
	N1/2/3	12 (70.6)	5	
M classification	M0	21 (45.7)	25	.037
	M1	4 (100)	0	
Stage	Stage I/II	2 (14.3)	12	.002
	Stage III/IV	23 (63.9)	13	
Recurrence	Negative	9 (31.0)	20	.002
	Positive	16 (76.2)	5	

^a^ Chi‐square test.

Kaplan–Meier analysis revealed that high TDO2 expression BC patients presented with shorter survival than those with low TDO2 expression BC patients (*p* = .009; Figure 1(D)). Kaplan–Meier analysis results from the BLCA dataset similarly showed unfavorable survival in BC patients with high TDO2 (Figure S1b). We then performed univariate and Cox regression analysis to investigate the possible role of TDO2 expression as an independent prognostic factor in BC. M classification and TDO2 expression were associated with worse survival in the univariate analysis (Table 2). However, only metastasis was recognized to be an independent prognostic factor in BC in the Cox analysis (Table 2).

**TABLE 2:** Univariate and multivariate Cox regression analysis of TDO2 expression and survival in bladder cancer

Characteristic	Univariate analysis		Multivariate analysis	
	HR (95% CI)	*p* value	HR (95% CI)	*p* value
Histological grade				
Low grade	1 (Ref)			
High grade	2.02 (0.87–4.69)	.102		
T classification				
T1/T2	1 (Ref.)			
T3/T4	1.72 (0.63–4.667)	.291		
N classification				
N0	1 (Ref.)			
N1/2	2.24 (0.96–5.28)	.063		
M classification				
M0	1 (Ref.)		1 (Ref.)	
M1	6.48 (2.05–20.44)	.001	4.13 (1.24–13.73)	.021
Stage				
Stage I/II	1 (Ref.)			
Stage III/IV	1.97 (0.66–5.858)	.222		
TDO2 expression				
Low	1 (Ref.)		1 (Ref.)	
High	3.15 (1.28–7.7)	.013	2.55 (0.98–6.62)	.054

Abbreviations: CI, confidence interval; HR, hazard ratio.

### 3.2 TDO2 expression is associated with basal‐type BC and CSC markers in BC

**FIGURE 2:**
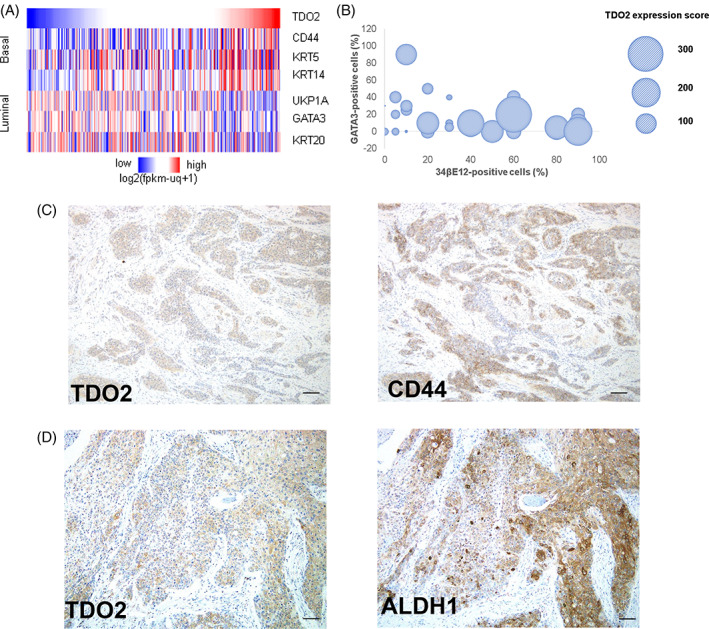
TDO2 expression is associated with basal‐type and CSCs markers in BC. (A) Heatmap showed upregulation of TDO2 gene expression associated with basal‐type BC in the TCGA dataset. (B) Bubble map shows that the expression of TDO2 correlated with 34βE12 and GATA3 staining in cells. (C, D) IHC analysis of TDO2, CD44, and ALDH1. Tumor cells presented co‐expressed TDO2 and CD44 staining (C) as well as TDO2 and ALDH1 staining (D). Scale bar, 50 μm

Recently, much large‐scale genome sequencing research in BC has revealed the molecular subtypes of BC shown to correlate with clinical progression, patient outcome, and response to treatment.^20^, ^21^, ^22^, ^23^ Although there are differences between the classification subtypes determined by each study, central to these classifications is the basal (expressed markers: CK5/6, CK14, CD44) BC type and the luminal (expressed markers: uroplakin, CK20, GATA3) BC type.^20^, ^21^, ^23^ We previously showed that TDO2 expression was associated with CD44 expression in esophagus squamous cell carcinoma.^13^ Hence, we considered that the expression of TDO2 might be related to the molecular subtype of BC. We first explored the correlation of TDO2 gene expression and basal or luminal gene expression markers in the BLCA dataset. As shown in Figure 2(A), the heatmap revealed that TDO2 gene expression correlated positively with basal gene expression markers (CD44, KRT5, KRT14) and inversely with luminal gene expression markers (UPK1A, GATA3, KRT20). To pursue this result, we downloaded and analyzed two other public gene sets from the GEO library (GSE31684 and GSE48277). TDO2 gene expression consistently showed a positive correlation with the basal markers and a negative correlation with the luminal markers (Figure S3a,b).

To determine the above gene expression results presented at the protein level, we performed IHC analysis of GATA3 (luminal marker) and 34βE12 (basal marker) in the 50 BC cases (Figure S3c). Interestingly, we found that TDO2 expression correlated with 34βE12 expression (rho = 0.494, *p* = 2.6274E‐04) but not with GATA3 expression (rho = 0. 253, *p* = .076) (Figure 2(B)). These data indicated that basal‐type BC highly expressed TDO2.

Previous results of BC genomic sequencing analysis revealed that the basal subtype of BC showed more stemness.^20^, ^21^, ^23^ The upregulated expression of two stem cell markers CD44 and ALDH1 was also shown to be associated with tumor stage, recurrence, and unfavorable prognosis of BC patients.^24^, ^25^ We then performed IHC analysis of CD44 and ALDH1 in the 50 BC cases. CD44 was strongly stained in the membrane of basal cells of normal urothelium, and ALDH1 showed strong cytoplasmic staining in BC cells. BC cells also presented co‐expressed TDO2 and CD44 staining as well as TDO2 and ALDH1 staining (Figure 2(C, D)). TDO2 expression was significantly correlated with CD44 and ALDH1 expression (Table 3).

**TABLE 3:** Correlation between TDO2 expression and CD44, ALDH1

		TDO2 expression, *n* (%)		*p*‐value^a^
		High	Low	
CD44	Positive	17 (63.0)	10	.047
	Negative	8 (34.8)	15	
ALDH1	Positive	16 (72.7)	6	.004
	Negative	9 (32.1)	19	

^a^ Chi‐square test.

### 3.3 TDO2 expression promotes cell proliferation in BC

**FIGURE 3:**
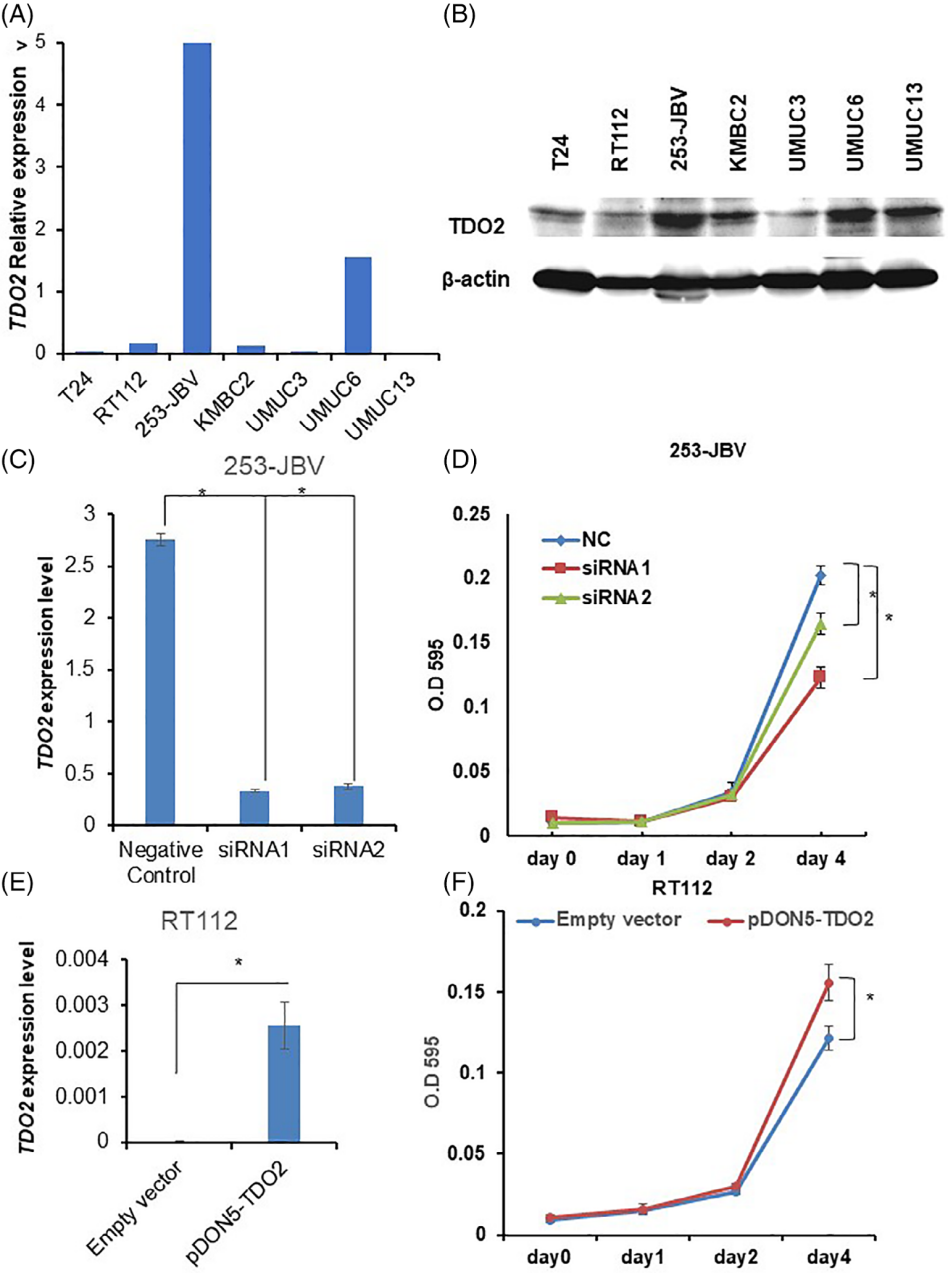
TDO2 promotes cell proliferation in BC cells. (A) TDO2 expression in seven BC cell lines by qRT‐PCR analysis. The data are displayed as mean ± SD, (*n* = 3). (B) Western blot analysis shows that 253‐JBV and UMUC6 cells highly expressed TDO2 among seven BC cell lines. (C) TDO2 mRNA levels expression in 253‐JBV cells transfected with two siRNAs targeting TDO2 (siRNA1, siRNA2) and negative control (NC) siRNA by qRT‐PCR analysis. The data are displayed as mean ± SD, (*n* = 3). (D) The proliferation of TDO2 siRNA‐transfected 253‐JBV cells was evaluated at 1, 2, and 4 days. NC: negative control. The error bars indicate SE, (*n* = 3). (E) TDO2 mRNA levels expression in RT112 cells transfected with TDO2 expression vector (pDON5‐TDO2) and empty vector by qRT‐PCR analysis. The data are displayed as mean ± SD, (*n* = 3). (F) The proliferation of RT112 transfection with TDO2 expression vector or empty vector was evaluated at 1, 2, and 4 days. The error bars indicate SE, (*n* = 3). *p < .01

We further investigated the effect of TDO2 knockdown on cell proliferation. Both qRT‐PCR and western blot analysis presented that 253‐JBV and UMUC6 cells highly expressed TDO2 (Figure 3(A, B)). Therefore, 253‐JBV and UMUC6 cells were selected for knockdown by siRNA. 253‐JBV and UMUC6 cells were transfected with two different siRNAs targeting TDO2. The level of TDO2 mRNA expression was significantly suppressed by siRNA1 and siRNA2 transfection (Figure 3(C), Figure S4a). As the effect of knockdown, 253‐JBV and UMUC6 cells transfected with TDO2 siRNA1and siRNA2 presented significantly lower cell proliferation compared with negative control siRNA‐transfected cells (*p* < .01) (Figure 3(D), Figure S4b).

To verify the biofunctional roles of TDO2 in BC, we generated a TDO2 overexpression vector. Then, we established stable TDO2 overexpression in RT112 and KMBC2 cells that showed a low level of TDO2 expression (Figure 3(A, B)). As shown in Figure 3(E) and Figure S5a, the expression levels of TDO2 were significantly increased in the transfected cells. Both BC cells (RT112 and KMBC2) transfected with TDO2 expression vector showed significantly induced cell growth rate compared with the cells transfected with empty vector (Figure 3(F), Figure S5b).

### 3.4 TDO2 promotes cell migration and invasiveness in BC

**FIGURE 4:**
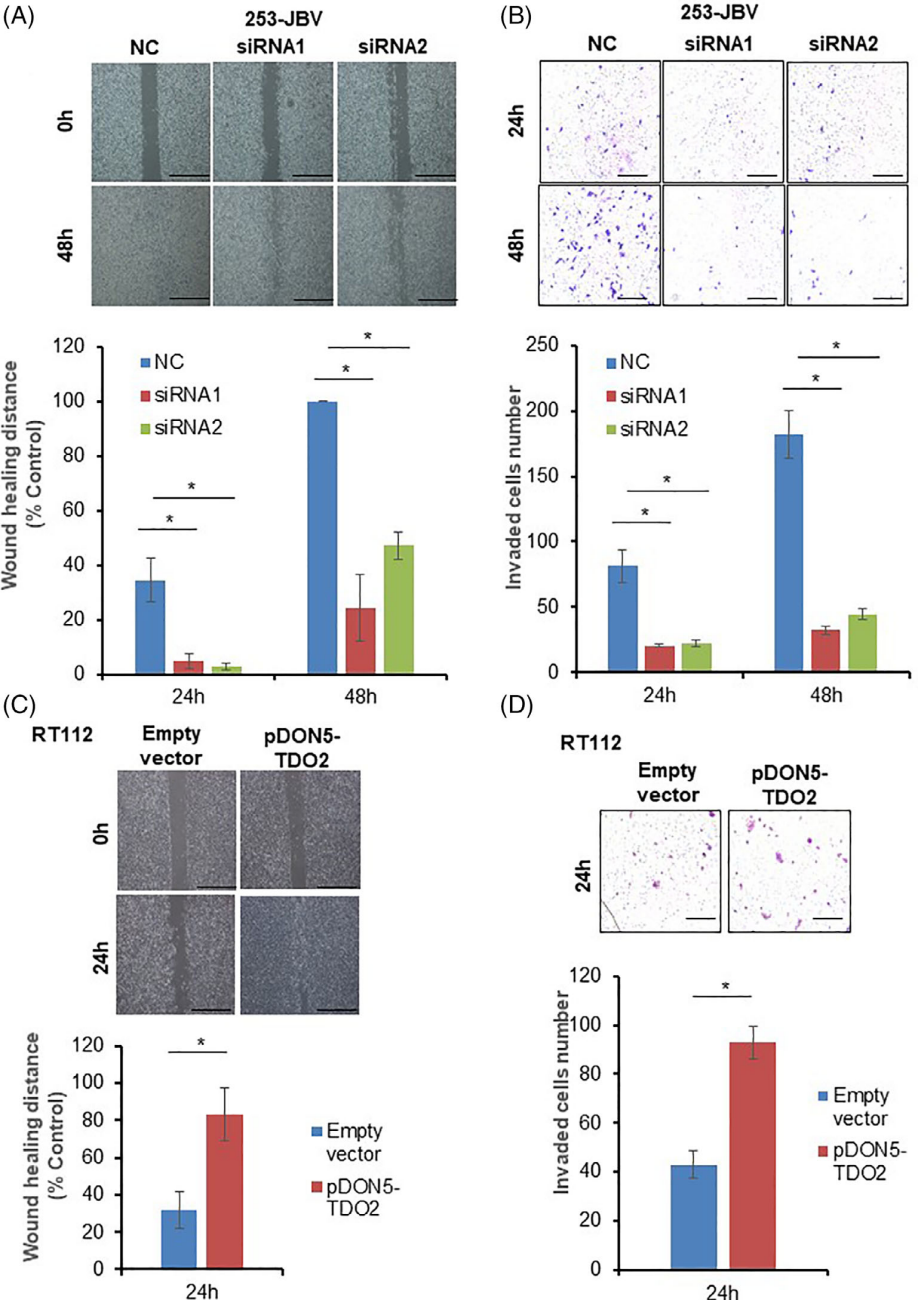
TDO2 promotes cell migration and invasiveness in BC. (A) Representative pictures of a cell migration assay in 253‐JBV cells transfected with two siRNAs targeting TDO2 (siRNA1, siRNA2) or siRNA negative control (NC). The migration of cells was captured at 0, 24, and 48 h after scratching. (B) Representative pictures of in vitro invasion assay in 253‐JBV cells transfected with two siRNAs targeting TDO2 (siRNA1, siRNA2) or siRNA negative control (NC). Quantification of the average number of invaded cells. The error bars indicate SE, (*n* = 3). Scale bar, 100 μm. (C) Representative pictures of a cell migration assay in RT112 cells transfected with TDO2 expression vector (pDON5‐TDO2) or empty vector. The migration of cells was captured at 0 and 24 h after scratching. (D) Representative images of in vitro invasion assay in RT112 cells transfected with TDO2 expression vector or empty vector. Quantification of the average number of invaded cells. The error bars indicate SE, (*n* = 3). Scale bar, 100 μm. *p < .01

High TDO2 expression was associated with tumor metastasis of BC patients. Hence, we evaluated the effect of TDO2 expression on the migration and invasiveness of BC cells. As shown in Figure 4(A, B) and Figure S4c,d, the cell migration and invasive activities of TDO2 siRNA1‐transfected and siRNA2‐transfected 253‐JBV and UMUC6 cells were lower than those of the negative‐control siRNA‐transfected 253‐JBV and UMUC6 cells (*p* < .05). Following these data, we performed cell migration assays and invasion assays using RT112 and KMBC2 cells with stable TDO2 overexpression. As expected, overexpression of TDO2 also promoted cell migration and invasiveness in the RT112 and KMBC2 cells (Figure 4(C, D), Figure S5c,d). These data indicated that TDO2 stimulates cell migration and invasive activities in BC cells.

### 3.5 TDO2 expression involved in spheroid formation

**FIGURE 5:**
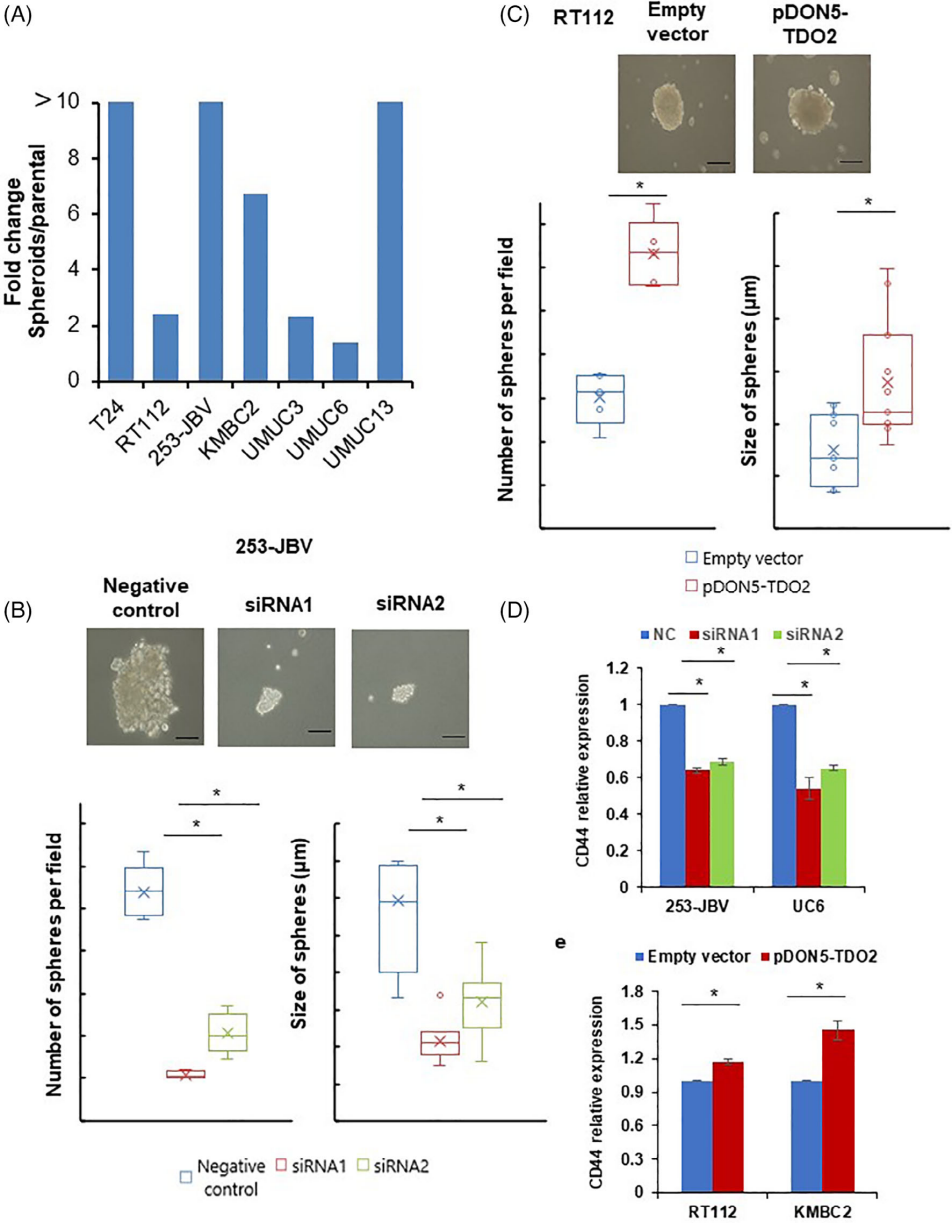
TDO2 expression involved in spheroid formation in BC cells. (A) Expression of TDO2 in spheroid formation cells and parental cells in seven BC cell lines by qRT‐PCR analysis. The data are displayed as mean ± SD, (*n* = 3). (B) The size and number of spheroid body‐forming cells from 253‐JBV cells transfected with two siRNAs targeting TDO2 (siRNA1, siRNA2) or negative control siRNA. (C) The size and number of spheroid body‐forming cells from RT112 cells transfected with TDO2 expression vector (pDON5‐TDO2) or empty vector. The error bars indicate SE, (*n* = 3). Scale bar, 10 μm. *p < .01. (D) CD44 mRNA levels expression in 253‐JBV and UMUC6 cells transfected with two siRNAs targeting TDO2 (siRNA1, siRNA2) or negative control siRNA (NC) by qRT‐PCR analysis. (E) CD44 mRNA levels expression in RT112 and KMBC2 cells transfected with TDO2 expression vector or empty vector by qRT‐PCR analysis. The data are displayed as mean ± SD, (*n* = 3). *p < .05

The IHC results revealed the correlation between TDO2 expression and cancer stem cell markers (CD44 and ALDH1) in BC. Spheroid colony formation assays are commonly used to investigate CSC features.^26^, ^27^ Thus, we measured the effect of TDO2 expression on spheroid colony formation. We generated spheroid body‐forming cells from seven BC cell lines. qRT‐PCR results displayed that the TDO2 mRNA level was significantly upregulated in the spheroid body‐forming cells (Figure 5(A)). We then evaluated the effect of TDO2 knockdown by siRNA transfection on spheroid colony number and size. Following day 15 after transfection, 253‐JBV and UMUC6 cells transfected with TDO2‐targeted siRNA showed significant reductions in both the spheroid colony size and number compared with siRNA negative control–transfected cells (Figure 5(B), Figure S6a).

We next evaluated the impact of TDO2 overexpression on spheroid colony formation of RT112 and KMBC2 cells. As shown in Figure 5(C) and Figure S6b, both BC cells (RT112 and KMBC2) with stable expression of TDO2 showed increases in both the spheroid colony number and size compared with the control cells. Interestingly, the 253‐JBV and UMUC6 siRNA1‐2‐transfected cells expressed a significantly lower level of CD44 expression, whereas RT112 and KMBC2 cells with stable expression of TDO2 upregulated the expression of CD44 (Figure 5(D)). Altogether, these data suggested that TDO2 is needed for spheroid colony formation and maintains cancer stemness in BC cells.

### 3.6 TDO2 induces resistance to cetuximab through integration of downstream signaling of EGFR pathway

**FIGURE 6:**
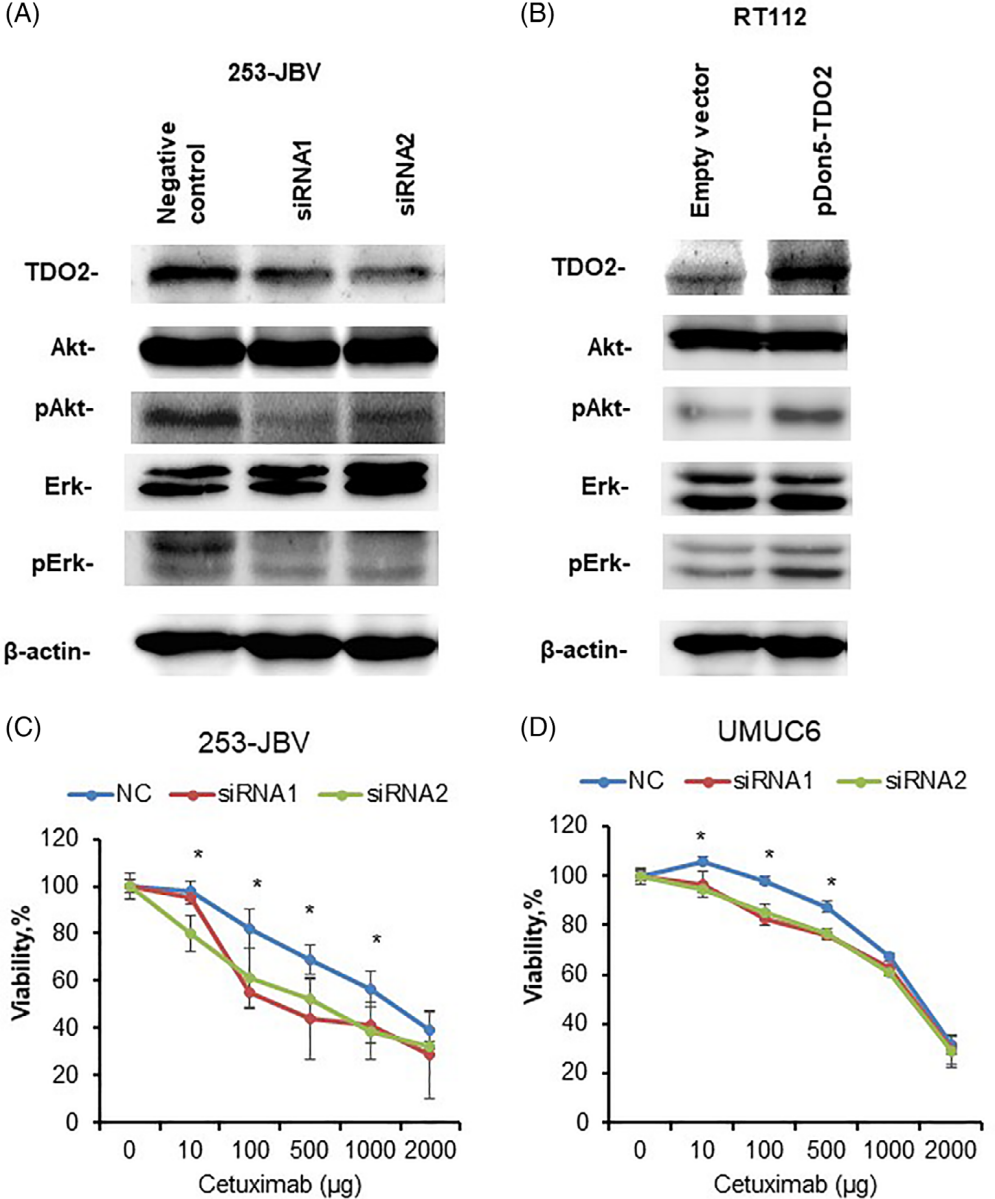
Effect of TDO2 on cetuximab sensitivity in BC cells. (A) Western blot analysis of TDO2, Erk1/2, phospho‐Erk1/2 (pErk1/2), Akt, and phospho‐Akt (pAkt) in 253‐JBV cells transfected with TDO2 siRNAs (siRNA1, siRNA2) or negative control siRNA (NC). β‐Actin was served as a loading control. (B) Western blot analysis of TDO2, Erk1/2, pErk1/2, Akt, and pAkt in RT112 cells transfected with TDO2 expression vector (pDON5‐TDO2) or empty vector. β‐Actin was served as a loading control. (C) Dose‐dependent effect of cetuximab on the viability of 253‐JBV cells transfected with TDO2 siRNAs or negative control siRNA. (D) Dose‐dependent effect of cetuximab on the viability of UMUC6 cells transfected with TDO2 siRNAs or negative control siRNA. The error bars indicate SE, (*n* = 3). *p < .01

Several lines of evidence revealed that EGFR may be a novel prognostic biomarker and molecular target in BC.^20^, ^21^, ^28^, ^29^ Recently, it was shown that aryl hydrocarbon receptor (AhR), activated by Kyn, a TDO2 metabolites product, bypasses EGFR to activate PI3K/AKT and MEK/ERK signaling.^30^, ^31^ Consequently, we evaluated the effect of TDO2 knockdown on the EGFR pathway. The knockdown of TDO2 expression in 253‐JBV and UMUC6 cells transfected with TDO2‐targeted siRNA was verified by Western blot analysis (Figure 6(A), Figure S7a). 253‐JBV and UMUC6 transfected with TDO2 siRNA1 and siRNA2 cells displayed the lower levels of phosphorylated Akt and Erk compared with that in control cells (Figure 6(A), Figure S7a). In contrast, the RT112 and KMBC2 cells with stable expression of TDO2 presented higher levels of phosphorylated Akt and Erk than control cells (Figure 6(B), Figure S7b). Hence, we examined the effect of TDO2 expression on the sensitivity to an EGFR‐targeted agent in BC cells. Cetuximab, a humanized monoclonal antibody against EGFR, binds to its extracellular domain to inhibit EGFR signaling and is used clinically for certain malignancies. As shown in Figure 6(C, D), inhibition of TDO2 by siRNA1 and siRNA2 in 253‐JBV and UMUC6 cells conferred sensitivity to cetuximab. Consequently, RT112 and KMBC2 with stable expression of TDO2 induced resistance to cetuximab (Figure S7c). However, knockdown of TDO2 did not affect the expression of Vimentin and E‐cadherin (Figure S8a,b). Taken together, these findings revealed that TDO2 can activate EGFR downstream signaling resulting in resistance to EGFR inhibitor in BC cells.

## 4 DISCUSSION

Innovative next genome sequencing research has clarified variable molecular subtypes of BC such as basal, luminal, mesenchymal, and Claudin‐low.^20^, ^21^, ^22^, ^23^ In this work, we showed that the TDO2 overexpression was correlated with advanced disease and unfavorable prognosis in BC patients. Notably, TDO2 was constitutively expressed in the basal BC subtype at both transcriptional and protein levels. TDO2 expression correlated with CD44 and ALDH1 expression in BCs, which was consistent with our previous study on esophagus squamous cell carcinoma.^13^ In breast cancer cells, TDO2 facilitates anoikis resistance,^12^ which is associated with CSC‐like cells.^12^, ^32^ Additionally, TDO2 persistently affected spheroid formation in BC cell lines in the knockdown and overexpression experiments, suggesting that the expression of TDO2 may take an essential role in BC stem cells.

TDO2 facilitates Kyn generation, which is a natural substance of AhR.^9^, ^33^ A comprehensive study in human brain tumors revealed that TDO2‐derived Kyn activates AhR in an autocrine/paracrine manner to promote tumor growth.^33^ Interestingly, kynurenine 3‐monooxygenase, an enzyme‐driven metabolic Kyn, acts as an oncogene in breast cancer by stimulating cells grown and invasiveness and enhances the stemness of cancer cells through beta‐catenin in breast cancer.^34^ Our results showed that TDO2 was involved in the proliferation, migration, and invasiveness of BC cells. Recently, a study which fully validated the expression of TDO2 by immunohistochemistry and in situ hybridization showed that liver cancer also highly expresses TDO2.^35^ These findings suggested that the TDO2‐Kyn‐AhR axis may have a crucial role in the tumorigenesis of cancer disease.

Currently, the first‐line treatment for advanced BC patients is platinum‐based chemotherapy.^4^, ^5^ In light of the approval of several immunotherapies such as atezolizumab and pembrolizumab (anti‐programmed cell death ligand‐1 antibodies), immune‐targeted therapy has been become a Category 2A recommendation in the NCCN Guidelines.^5^ A previous report showed that TDO2‐expressing tumors induced acquired immune tolerance.^10^ IDO1/TDO2 inhibitor has been used in human phase I clinical trials.^36^ In the present study, we showed that TDO2 could have an essential role in promoting cell survival, cell migration, and invasion of BC cells. Additionally, combining the IDO1 inhibitor with nivolumab is safe and improves response rates in bladder and cervical cancer patients.^37^ Interestingly, IDO1 inhibition was also shown to inhibit cell growth, migration, and invasion in BC cells.^38^ Taken together, TDO2/IDO1 may serve as an ideal target for the development of targeted therapy.

The EGFR signaling pathway is the most well‐known pathway playing pivotal roles in oncogenesis. Transcriptome analysis pointed out that up to 11% of MIBC patients present with EGFR amplification.^20^, ^21^ EGFR is overexpressed in up to 74% of BC tissue specimens.^39^ Therefore, EGFR could be a potential therapeutic target for BC. There are two main agents against EGFR: monoclonal antibodies against the EGFR including cetuximab and tyrosine kinase inhibitors like gefitinib and erlotinib.^40^ EGFR inhibitors inhibit phosphorylation of EGFR, which consequently downregulates phosphorylated AKT and ERK. Therefore, tumor cells begin to harbor resistance to tyrosine kinase inhibitors and anti‐EGFR monoclonal antibodies by modulating MEK and ERK signaling.^41^ One particular finding in the present work is that ERK and AKT phosphorylation were dramatically inactivated by the knockdown of, and activated by the overexpression of, TDO2 in BC. Furthermore, we showed that inhibition of TDO2 made BC cells sensitive to cetuximab and forced expression of TDO2 made BC cells resistant to cetuximab. Our data corroborate the research findings in non‐small‐cell lung cancer, that is, activation of AhR by a TDO2 metabolite, Kyn, promotes resistance to EGFR inhibitors by rescuing PI3K/AKT and MEK/ERK signaling.^31^ Together, these findings indicated that TDO2 may take part in the activation of EGFR downstream signaling and be involved in the resistance of TKIs in BC.

In conclusion, this study showed that TDO2 overexpression was related to disease progression and unfavorable prognosis in BC patients. In vitro experiments revealed that TDO2 participated in cancer cell proliferation, migration, invasion, and spheroid body formation in BC cells. TDO2 recruited ERK and AKT phosphorylation and made BC cells resistant to cetuximab. Our results demonstrate that TDO2 could be a potential marker for targeted therapy in BC.

## CONFLICT OF INTEREST

The authors report no potential conflicts of interest.

## AUTHORS' CONTRIBUTIONS

All authors had full access to the data in the study and take responsibility for the integrity of the data and the accuracy of the data analysis. *Data Curation*, Q.T.P., D.T.; *Methodology*, Q.T.P., W.Y.; *Investigation*, Q.T.P., W.Y.; *Formal Analysis*, Q.T.P., D.T., W.Y.; *Resources*, D.T., S.A., K.H., T.B., Y.S., W.Y.; *Writing ‐ Original Draft*, Q.T.P., W.Y.; *Writing ‐ Review & Editing*, Q.T.P., W.Y.; *Visualization*, Q.T.P., S.A., K.H., T.B., Y.S., N.S., K.S., N.O., W.Y.; *Supervision*, D.T., W.Y.; *Funding Acquisition*, W.Y.; *Validation*, Q.T.P., S.A., K.H., N.S., K.S., N.O., W.Y.; *Software*, Q.T.P., Y.S., *Project Administration*, W.Y.

## ETHICAL STATEMENT

This study was approved by the Ethical Committee for Human Genome Research of Hiroshima University, Hiroshima, Japan (approval no. IRINHI66).

## Supporting information


**Data S1.** Supporting information.

**Data S2.** Supporting information.

## Data Availability

An online analytical web‐based tool, https://xenabrowser.net/, was conducted to evaluate the mRNA expression of TDO2. The public datasets (GSE31684 and GSE48277) were downloaded from Gene Expression Omnibus (GEO) database, https://www.ncbi.nlm.nih.gov/geo/.
