# Supplementary material for: TDO2 overexpression correlates with poor prognosis, cancer stemness, and resistance to cetuximab in bladder cancer
Source: Cancer Rep (Hoboken). 2021 Jun 7;4(6):e1417. doi: 10.1002/cnr2.1417 (PMC8714553; doi:10.1002/cnr2.1417)
Supplement: Supplementary file 1 — Data S1. Supporting information. [file CNR2-4-e1417-s001.docx]

**Supplementary Materials and Methods**

*Primer sequences*

TDO2 were forward, 5′-CGG TGG TTC CTC AGG CTA TC-3′ and reverse, 5′-CTT CGG TAT CCA GTG TCG GG-3′.

CD44 were forward, 5′-TAC AGC ATC TCT CGG ACG GA-3′ and reverse, 5′-CAC CCC TGT GTT GTT TGC TG-3′.

ACTB were forward, 5′-CTG TCT GGC GGC ACC ACC AT-3′ and reverse, 5′-GCA ACT AAG TCA TAG TCC GC-3′.

*RNAi*

Short interfering RNA (siRNA) oligonucleotides targeting *TDO2* and a negative control were purchased from Invitrogen (Carlsbad, CA, USA). We used two different *TDO2* siRNA oligonucleotide sequences: siRNA1: 5′-AUA CCU UGU ACC UAU CAC UCA CAG U-3′ and siRNA2: 5′-CCC GAC ACU GGA UAC CGA AGA UGA A-3′. Transfection was performed using Lipofectamine RNAiMAX (Invitrogen) as previously described.[^13^](#_ENREF_13)

*Expression vector*

For constitutive expression of TDO2, cDNA was PCR amplified and subcloned into pDON5-Neo (Invitrogen). Primer sequences for the full-length amplification of TDO2 were forward TDO2-ApaI, 5′-CGC GGG CCC ATG AGT GGG TGC CCA TTT TTA-3′ and reverse TDO2-BamHI: 5′-CGC GGA TCC ATC TGA TTC ATC ACT GCT GA-3′. The pDON5-Neo-TDO2 expression vector was transfected into BC cell lines with FuGENE6 (Promega) according to the manufacturer’s instructions. Cells with stable TDO2 expression were selected after 2 weeks of culture with 400 µg G418 (Life Technologies, CA, USA).

*Cell proliferation assays*

We performed 3-(4,5-dimethylthiazol-2-yl)-2,5-diphenyltetrazolium bromide (MTT) assays. The cells were seeded at a density of 3000 cells per well in 96-well plates. Cell growth was examined after 1, 2, and 4 days. Three independent experiments were performed. The mean and standard error (SE) were calculated for each experiment.

*Cell migration and invasion assays*

To monitor cell migration, wound healing assays were carried out with the ibidi Culture-Insert 2 well (ibidi GmbH, Gräfelfing, Germany). Cells were suspended at 10^6^ cells/ml in RPMI medium, and 70 µl of cells were applied into each well. After 24 hours, the insert was gently removed, and the cells were cultured with serum-free RPMI medium. Images were obtained at the appropriate times using phase contrast microscopy.

Modified Boyden chamber assays were performed to examine cell invasiveness. Cells were plated at 20,000 cells per well in RPMI 1640 medium plus 1% serum in the upper chamber of a Transwell Insert (8 µm pore diameter; Chemicon, Temecula, CA, USA) coated with Matrigel. Medium containing 10% serum was added in the bottom chamber. After 2 days, cells in the upper chamber were removed by scraping, and the cells remaining on the lower surface of the insert were stained with CyQuant GR dye (Chemicon) to assess the number of cells. Three independent experiments were carried out. The mean and SE were calculated for each of the experiments.

*Spheroid colony formation assay*

For the generation of spheres, 2000 cells were plated in each well of 24-well ultra-low-attachment plates (Corning) containing mTeSR medium (STEMCELL Technologies Inc., Cambridge, MA, USA). The plates were incubated at 37°C in a 5% CO_2_ incubator for 15 days. Sphere number and size were determined and counted under a microscope.

*Statistical methods*

Associations between clinicopathological parameters and TDO2 expression were analyzed by Chi-squared test. Spearman rank correlation was used to analyze the correlation between TDO2 expression and GATA3 and 34βE12 expression. Kaplan-Meier survival curves were constructed for patients with high and low expression of TDO2. Survival rates were compared between the high and low TDO2 expression groups. Differences between survival curves were tested for statistical significance by a log-rank test. Differences between the two groups (*TDO2* siRNA-transfected cells and negative control siRNA-transfected cells or pDON5-Neo-TDO2 expression vector and pDON5 empty vector) were tested by Student *t*-test. A *p* value of <0.05 was considered to indicate statistical significance.
