# Supplementary material for: TDO2 overexpression correlates with poor prognosis, cancer stemness, and resistance to cetuximab in bladder cancer
Source: Cancer Rep (Hoboken). 2021 Jun 7;4(6):e1417. doi: 10.1002/cnr2.1417 (PMC8714553; doi:10.1002/cnr2.1417)
Supplement: Supplementary file 2 — Data S2. Supporting information. [file CNR2-4-e1417-s002.pptx]

## Slide 1
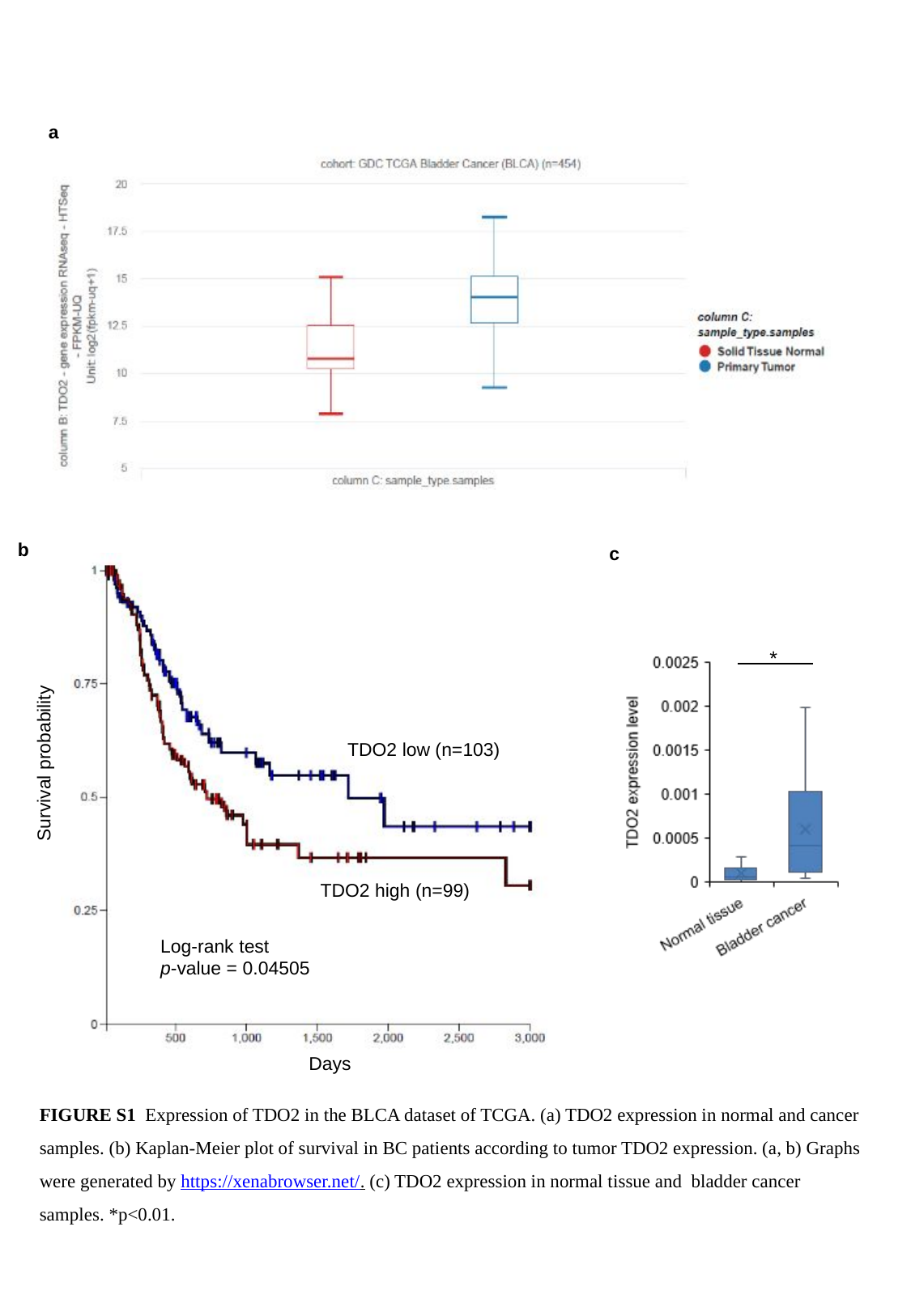

a
b
c
*
Survival probability
TDO2 low (n=103)
TDO2 high (n=99)
Log-rank test
p-value = 0.04505
Days
FIGURE S1 Expression of TDO2 in the BLCA dataset of TCGA. (a) TDO2 expression in normal and cancer samples. (b) Kaplan-Meier plot of survival in BC patients according to tumor TDO2 expression. (a, b) Graphs were generated by https://xenabrowser.net/. (c) TDO2 expression in normal tissue and bladder cancer samples. *p<0.01.

## Slide 2
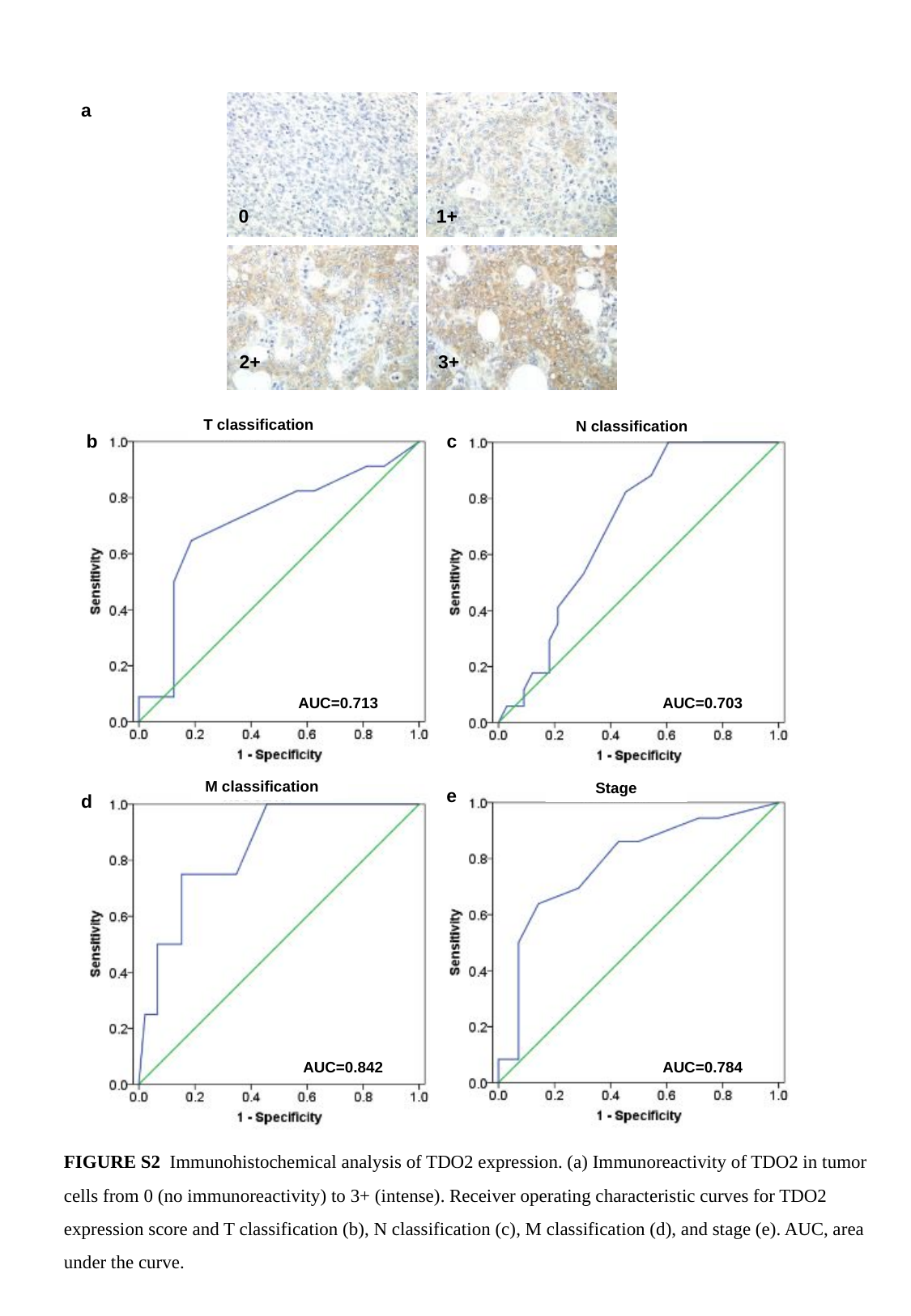

a
0
1+
2+
3+
T classification
N classification
AUC=0.713
AUC=0.703
AUC=0.784
AUC=0.842
b
c
M classification
Stage
e
d
FIGURE S2 Immunohistochemical analysis of TDO2 expression. (a) Immunoreactivity of TDO2 in tumor cells from 0 (no immunoreactivity) to 3+ (intense). Receiver operating characteristic curves for TDO2 expression score and T classification (b), N classification (c), M classification (d), and stage (e). AUC, area under the curve.

## Slide 3
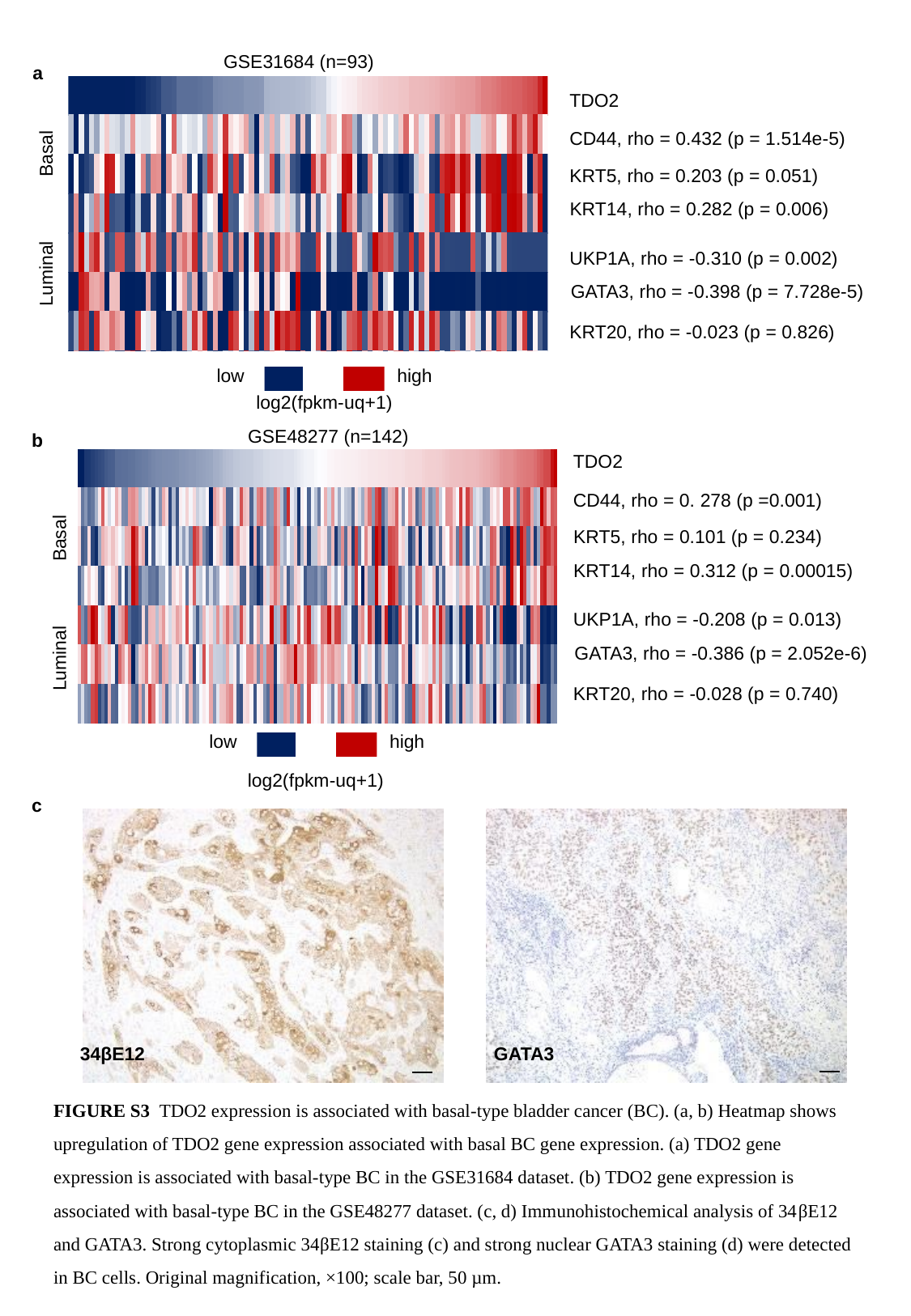

GSE31684 (n=93)
a
TDO2
CD44, rho = 0.432 (p = 1.514e-5)
Basal
KRT5, rho = 0.203 (p = 0.051)
KRT14, rho = 0.282 (p = 0.006)
UKP1A, rho = -0.310 (p = 0.002)
Luminal
GATA3, rho = -0.398 (p = 7.728e-5)
KRT20, rho = -0.023 (p = 0.826)
high
low
log2(fpkm-uq+1)
GSE48277 (n=142)
b
TDO2
CD44, rho = 0. 278 (p =0.001)
KRT5, rho = 0.101 (p = 0.234)
Basal
KRT14, rho = 0.312 (p = 0.00015)
UKP1A, rho = -0.208 (p = 0.013)
GATA3, rho = -0.386 (p = 2.052e-6)
Luminal
KRT20, rho = -0.028 (p = 0.740)
high
low
log2(fpkm-uq+1)
c
34βE12
GATA3
FIGURE S3 TDO2 expression is associated with basal-type bladder cancer (BC). (a, b) Heatmap shows upregulation of TDO2 gene expression associated with basal BC gene expression. (a) TDO2 gene expression is associated with basal-type BC in the GSE31684 dataset. (b) TDO2 gene expression is associated with basal-type BC in the GSE48277 dataset. (c, d) Immunohistochemical analysis of 34βE12 and GATA3. Strong cytoplasmic 34βE12 staining (c) and strong nuclear GATA3 staining (d) were detected in BC cells. Original magnification, ×100; scale bar, 50 µm.

## Slide 4
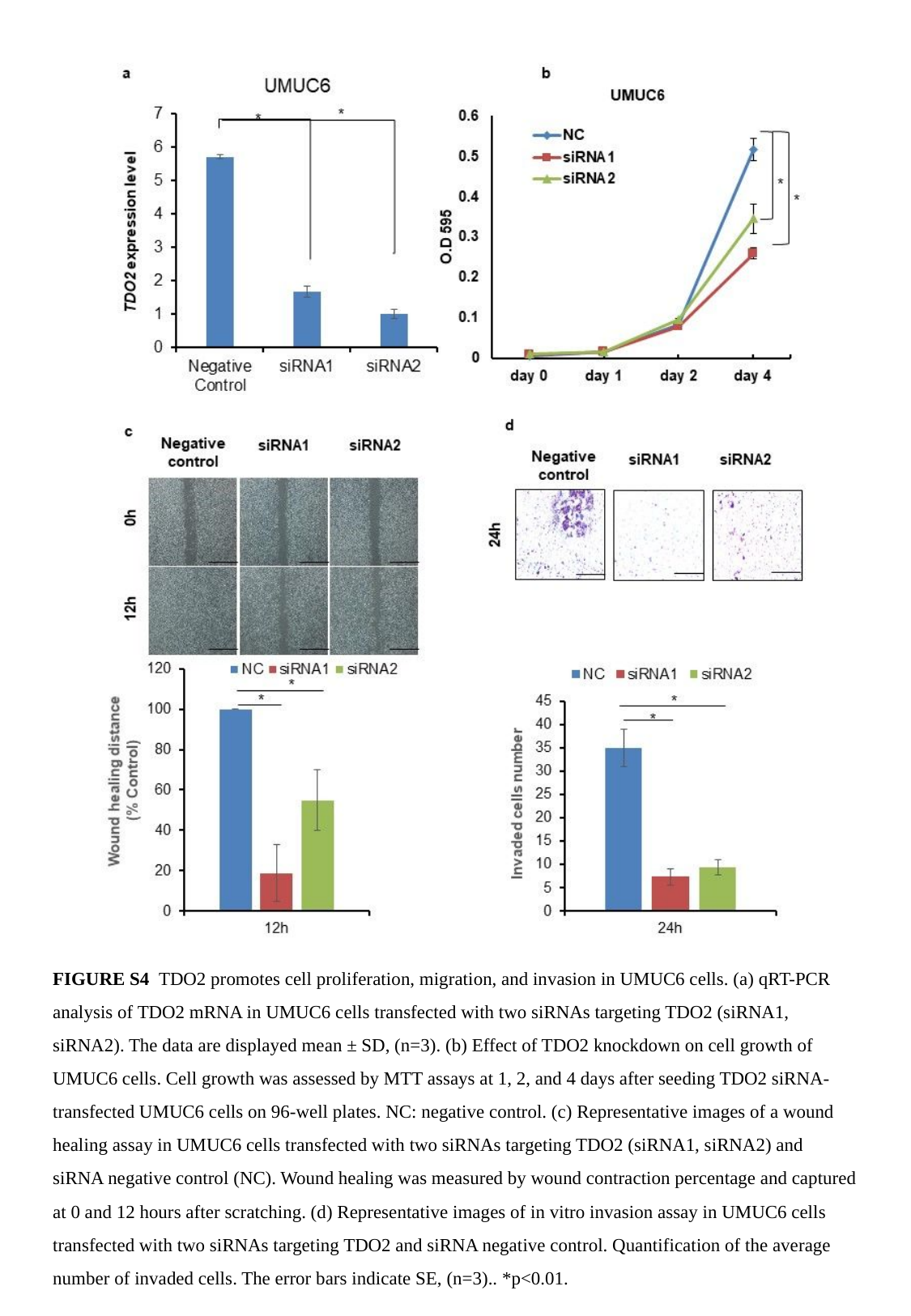

FIGURE S4 TDO2 promotes cell proliferation, migration, and invasion in UMUC6 cells. (a) qRT-PCR analysis of TDO2 mRNA in UMUC6 cells transfected with two siRNAs targeting TDO2 (siRNA1, siRNA2). The data are displayed mean ± SD, (n=3). (b) Effect of TDO2 knockdown on cell growth of UMUC6 cells. Cell growth was assessed by MTT assays at 1, 2, and 4 days after seeding TDO2 siRNA-transfected UMUC6 cells on 96-well plates. NC: negative control. (c) Representative images of a wound healing assay in UMUC6 cells transfected with two siRNAs targeting TDO2 (siRNA1, siRNA2) and siRNA negative control (NC). Wound healing was measured by wound contraction percentage and captured at 0 and 12 hours after scratching. (d) Representative images of in vitro invasion assay in UMUC6 cells transfected with two siRNAs targeting TDO2 and siRNA negative control. Quantification of the average number of invaded cells. The error bars indicate SE, (n=3).. *p<0.01.

## Slide 5
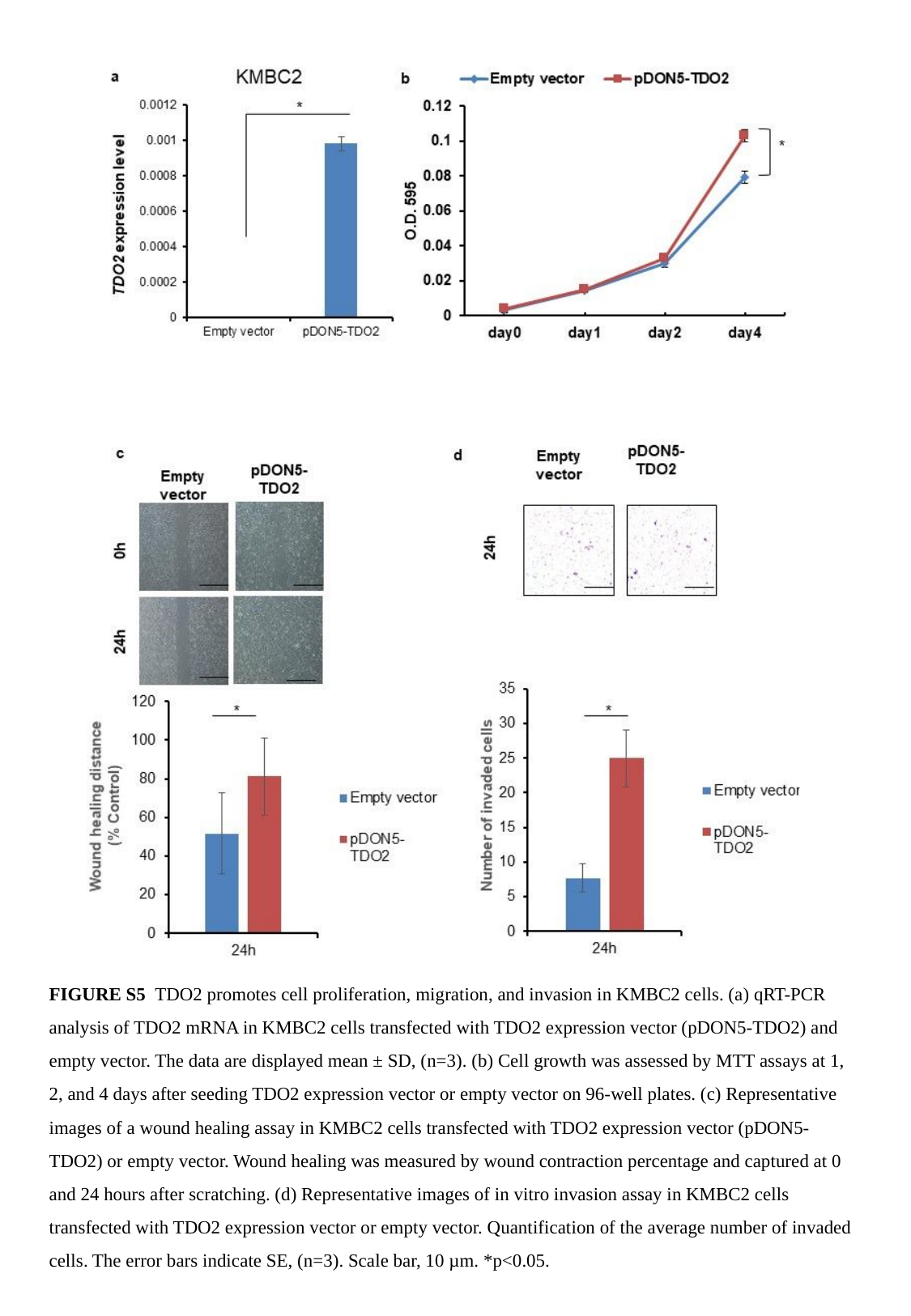

FIGURE S5 TDO2 promotes cell proliferation, migration, and invasion in KMBC2 cells. (a) qRT-PCR analysis of TDO2 mRNA in KMBC2 cells transfected with TDO2 expression vector (pDON5-TDO2) and empty vector. The data are displayed mean ± SD, (n=3). (b) Cell growth was assessed by MTT assays at 1, 2, and 4 days after seeding TDO2 expression vector or empty vector on 96-well plates. (c) Representative images of a wound healing assay in KMBC2 cells transfected with TDO2 expression vector (pDON5-TDO2) or empty vector. Wound healing was measured by wound contraction percentage and captured at 0 and 24 hours after scratching. (d) Representative images of in vitro invasion assay in KMBC2 cells transfected with TDO2 expression vector or empty vector. Quantification of the average number of invaded cells. The error bars indicate SE, (n=3). Scale bar, 10 µm. *p<0.05.

## Slide 6
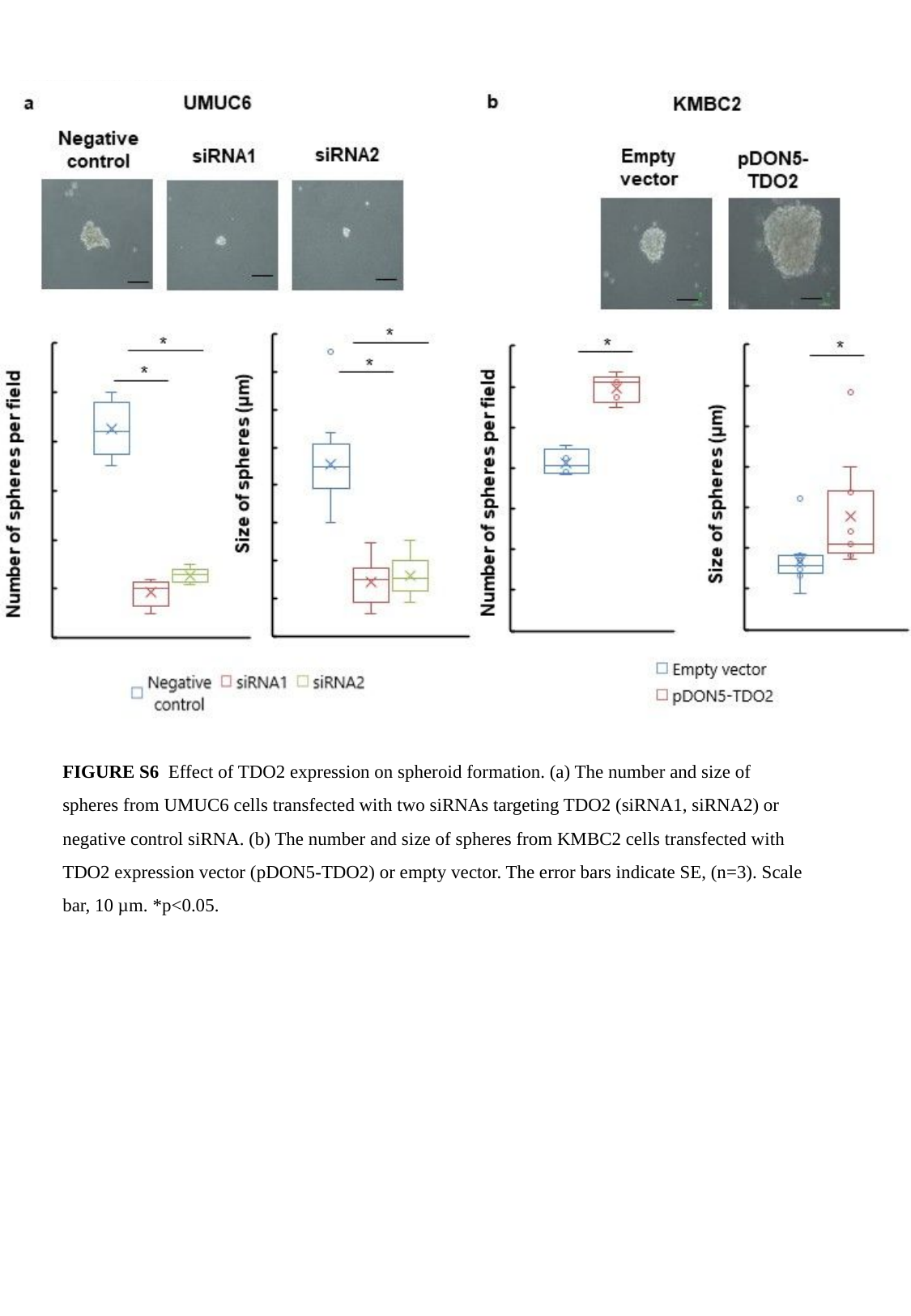

FIGURE S6 Effect of TDO2 expression on spheroid formation. (a) The number and size of spheres from UMUC6 cells transfected with two siRNAs targeting TDO2 (siRNA1, siRNA2) or negative control siRNA. (b) The number and size of spheres from KMBC2 cells transfected with TDO2 expression vector (pDON5-TDO2) or empty vector. The error bars indicate SE, (n=3). Scale bar, 10 µm. *p<0.05.

## Slide 7
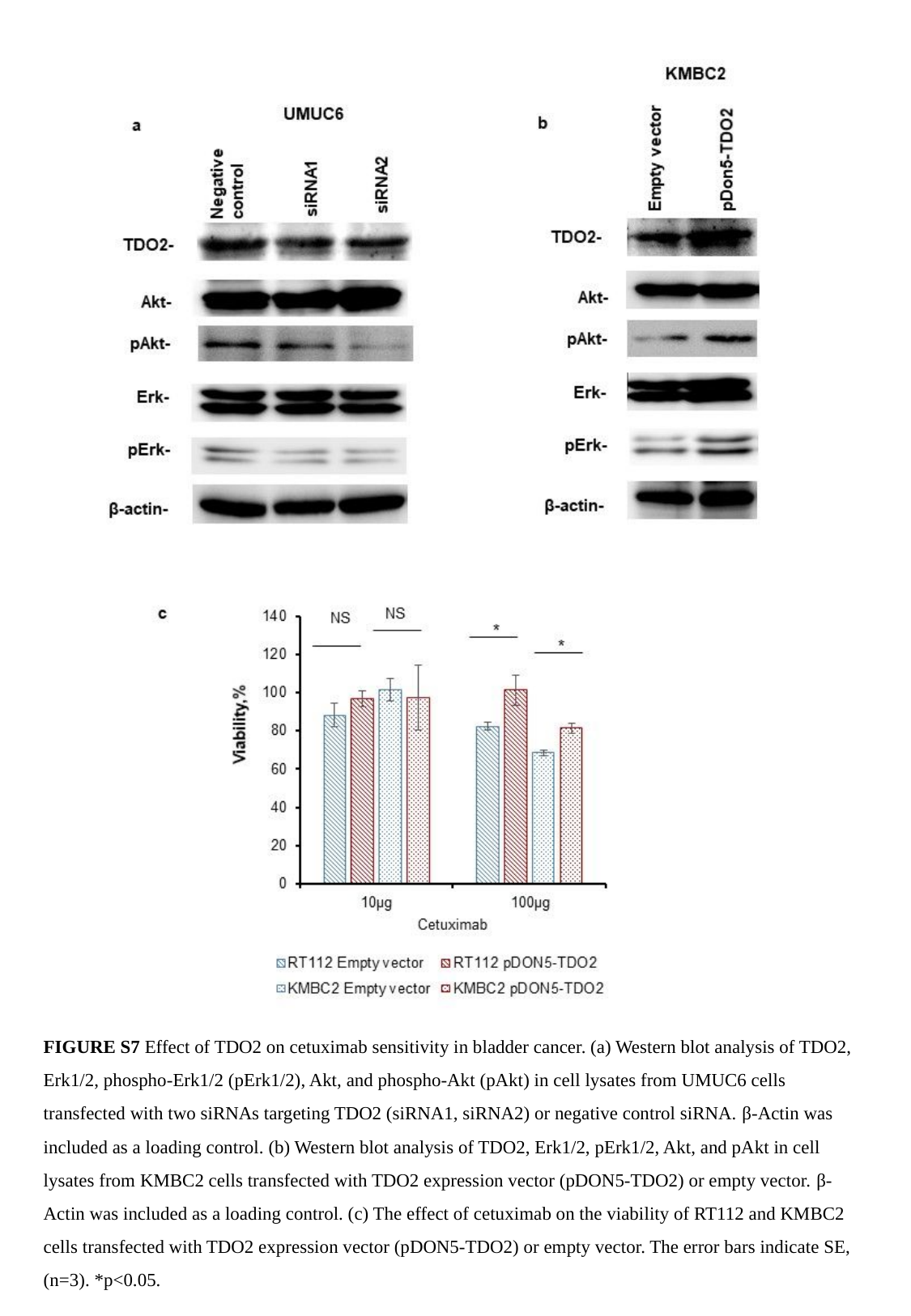

FIGURE S7 Effect of TDO2 on cetuximab sensitivity in bladder cancer. (a) Western blot analysis of TDO2, Erk1/2, phospho-Erk1/2 (pErk1/2), Akt, and phospho-Akt (pAkt) in cell lysates from UMUC6 cells transfected with two siRNAs targeting TDO2 (siRNA1, siRNA2) or negative control siRNA. β-Actin was included as a loading control. (b) Western blot analysis of TDO2, Erk1/2, pErk1/2, Akt, and pAkt in cell lysates from KMBC2 cells transfected with TDO2 expression vector (pDON5-TDO2) or empty vector. β-Actin was included as a loading control. (c) The effect of cetuximab on the viability of RT112 and KMBC2 cells transfected with TDO2 expression vector (pDON5-TDO2) or empty vector. The error bars indicate SE, (n=3). *p<0.05.

## Slide 8
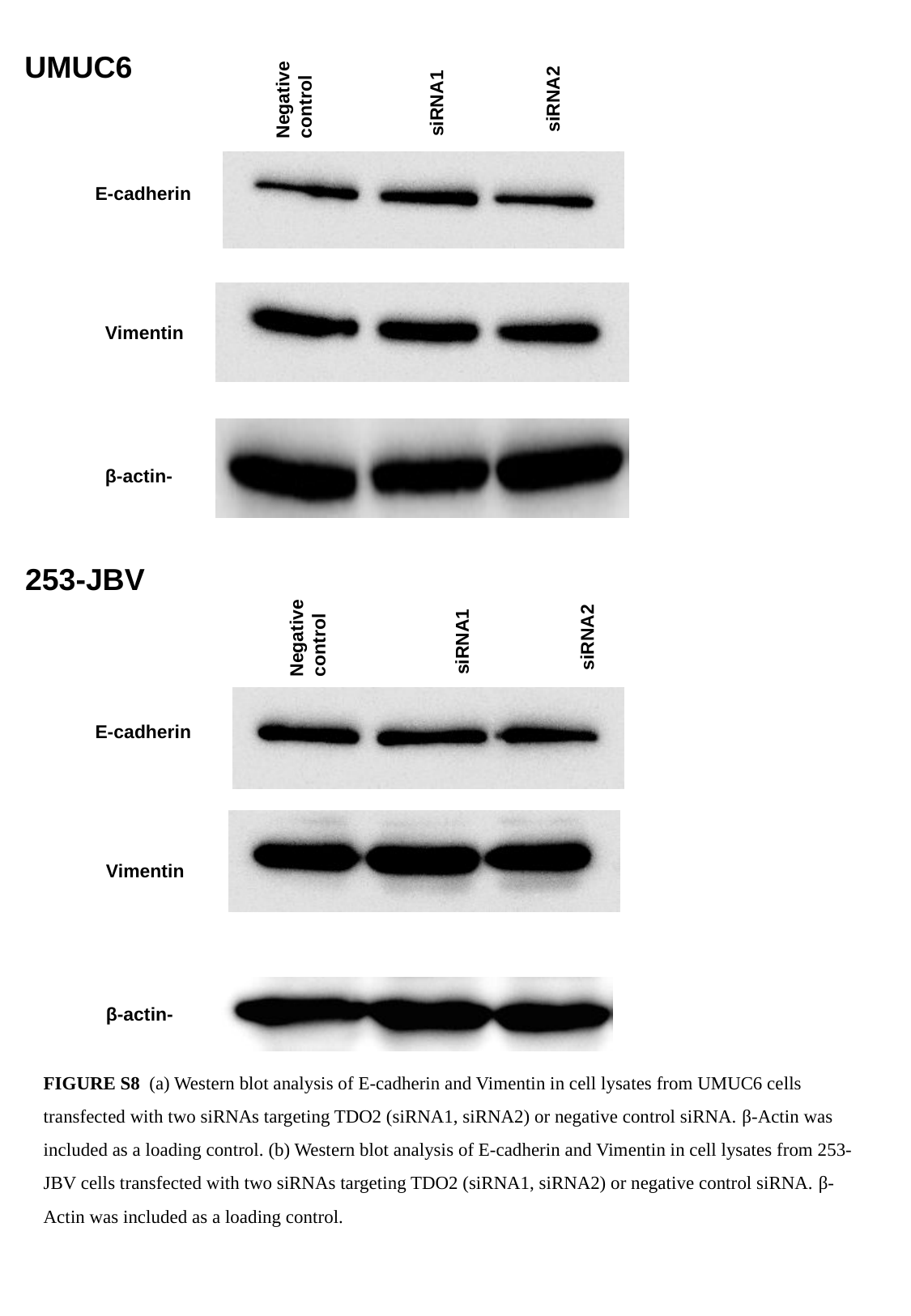

Negative
control
siRNA2
siRNA1
E-cadherin
Vimentin
β-actin-
UMUC6
Negative
control
siRNA2
siRNA1
E-cadherin
Vimentin
β-actin-
253-JBV
FIGURE S8 (a) Western blot analysis of E-cadherin and Vimentin in cell lysates from UMUC6 cells transfected with two siRNAs targeting TDO2 (siRNA1, siRNA2) or negative control siRNA. β-Actin was included as a loading control. (b) Western blot analysis of E-cadherin and Vimentin in cell lysates from 253-JBV cells transfected with two siRNAs targeting TDO2 (siRNA1, siRNA2) or negative control siRNA. β-Actin was included as a loading control.
